# Evaluation of the EUCAST Rapid Antimicrobial Susceptibility Test for *Enterobacterales*-Containing Blood Cultures in China

**DOI:** 10.1128/jcm.02559-21

**Published:** 2022-03-31

**Authors:** Yuzhang Shan, Bijie Hu, Wei Guo, Beili Wang, Chunmei Zhou, Shenlei Huang, Na Li

**Affiliations:** a Department of Laboratory Medicine, Zhongshan Hospital of Fudan University, Shanghai, China; b Department of Infectious Diseases, Zhongshan Hospital of Fudan University, Shanghai, China; Johns Hopkins

**Keywords:** bloodstream infection, *Enterobacterales*, disk diffusion, rapid antimicrobial susceptibility testing, ceftazidime-avibactam

## Abstract

Bloodstream infection (BSI) is defined by the presence of microbes in the bloodstream and has high mortality. Early antimicrobial therapy is key to treating BSI patients. Because of potential antimicrobial resistance, rapid evaluation for the most suitable antimicrobial therapy is important for appropriate treatment. In China, the current workflow of microbiological diagnosis in BSI involves blood culture, species identification, and antimicrobial susceptibility testing, which takes around 3 days. However, this delay could lead to worse symptoms. To rapidly and accurately assess antimicrobial susceptibility, in this study, we applied EUCAST rapid antimicrobial susceptibility testing (RAST) to determine the antimicrobial susceptibilities of the most frequently detected *Enterobacterales* sampled in China, including Escherichia coli and Klebsiella pneumoniae. Based on EUCAST guidelines, we evaluated its efficiencies with six commercially available antimicrobials, including imipenem (10 μg), meropenem (10 μg), ciprofloxacin (5 μg), levofloxacin (5 μg), amikacin (30 μg), and trimethoprim-sulfamethoxazole (1.25/23.75 μg), with bacterium-spiked blood cultures. In addition, we developed potential breakpoints for a recently introduced antimicrobial, 30/20 μg ceftazidime-avibactam, which has high potential for treating multidrug-resistant *Enterobacterales*. Our results showed that EUCAST RAST is a reliable method for rapidly determining the antimicrobial susceptibilities of BSI-causing bacteria in China, with an overall categorical agreement rate at 8 h of ≥90%. The breakpoints developed in this study can categorize the isolates sampled in this study with an accuracy of 93%. Results from our experiments can be applied to clinically determine the microbial susceptibility of BSI-causing bacteria within 8 h and benefit clinical diagnostics for BSI patients.

## INTRODUCTION

Bloodstream infections (BSI) are infectious diseases defined by the presence of microbes in the bloodstream, for example, the most often detected members of the *Enterobacterales*, such as Escherichia coli and Klebsiella pneumoniae (1). BSI can cause a series of systematic symptoms, such as sepsis, septic shock, and organ dysfunction with high mortality (2). To clinically treat BSI, immediate treatment with antimicrobials has been often used to eliminate infecting microbes and relieve related symptoms. However, since antimicrobials are usually used empirically without correlation with potential antimicrobial resistance, studies have reported enhanced risk of BSI-related morbidity and mortality resulting from misuse of antimicrobials, especially for severely ill or septic patients (3–6). One of the clinical solutions is to evaluate the antimicrobial susceptibility with appropriate antimicrobial susceptibility testing (AST) and molecular assays so that the most efficient antimicrobials can be administered (1).

The evaluation of antimicrobial susceptibility for infecting microbes in BSI relies on blood cultures and conventional laboratory methods. These methods usually require 3 days to determine the organisms’ susceptibility to available drugs, which leads to a delay before administration of antimicrobial treatments (1, 7). This delay has been the major obstacle for clinicians in curing patients. To overcome this obstacle, a rapid antimicrobial susceptibility test (RAST) has been developed by the European Committee on Antimicrobial Susceptibility Testing (EUCAST), which provides a rapid evaluation of the antimicrobial susceptibility within 8 h. By January 2021, EUCAST had provided practical guidance in determining the antimicrobial susceptibility for 13 different antimicrobial agents in European countries treating E. coli and K. pneumoniae (8, 9). However, this rapid evaluation method has rarely been used in countries outside Europe, for example, China and the United States, where BSI also cause severe health issues (2, 10).

One of the most intractable issues in treating BSI in China is the presence of antimicrobial resistance in infecting microbes acquired from different sources (10). Studies have shown that the case number of multidrug-resistant microbes detected from BSI in China has increased remarkably since 2005 (11). The antimicrobial resistance shows region-specific patterns both in China and at the global scale (12). For example, Shanghai, the economic center of China, has been reported to have the highest prevalence of antimicrobial resistance (11). In antimicrobial treatments in China, the most frequently administered antimicrobials for susceptible and multidrug-resistant microbial infections are quinolones and carbapenems, respectively. Although these two types of antimicrobials are able to treat most microbes, bacteria that are resistant to these antimicrobials have been detected with high prevalence in many areas in China (13). In 2019, a novel antibiotic, ceftazidime-avibactam, was introduced by the China National Medical Products Administration and has shown significant efficacy in treating 75% of carbapenem-resistant *Enterobacterales* (CRE) in China (14). Although EUCAST has provided RAST guidelines for ceftazidime-avibactam, their concentration setup is different from that of test kits available in non-European countries, rendering these guidelines unsuitable for use in clinical settings. Under the circumstances where a rapid RAST method is required in China, a clinical guideline for this newly introduced antibiotic is needed.

In this study, we investigated whether the EUCAST RAST method is applicable to evaluate antimicrobial resistance in BSI-related microbes in China. We conducted a series of experiments to compare its efficiency with that of seeded blood cultures containing *Enterobacterales* for six conventional antimicrobials. In addition, we also applied the EUCAST RAST method to the novel antibiotic ceftazidime-avibactam at the disk concentration (30/20 μg) recommended by the Clinical and Laboratory Standards Institute (CLSI). We developed preliminary breakpoints for the EUCAST RAST method with ceftazidime-avibactam in BSI treatment. To further evaluate its accuracy, we also characterized the presence of antimicrobial resistance genes in the genomes of all CRE isolates in this study. Results from this study can improve the practicality of the EUCAST RAST method in China and provide practical guidelines for the use of novel antibiotics for clinicians treating BSI patients.

## MATERIALS AND METHODS

### Bacterial source.

In this study, 72 Klebsiella pneumoniae and 43 Escherichia coli isolates were randomly collected from clinical samples in intensive care units (ICU) in Fudan University Zhongshan Hospital from 1 December 2017 to 31 December 2020. Once isolates had been collected, Vitek MS (bioMérieux, France) was used to obtain species-specific mass spectra, which were then compared with the IVD 3.0 database for species identification. After the species were confirmed, all isolates were frozen at −20°C for future experiments.

### Commercial antimicrobial susceptibility test.

Six conventional and the most commonly used antimicrobials in China were selected in this study, including amikacin (30 μg), ciprofloxacin (5 μg), levofloxacin (5 μg), imipenem (10 μg), meropenem (10 μg), and trimethoprim-sulfamethoxazole (1.25/23.75 μg). We first applied a traditional antimicrobial susceptibility test to determine the antimicrobial susceptibility using Vitek 2 (bioMérieux, France). For each bacterial isolate, 145 μL of bacterial suspension at 1.5 × 10^8^ CFU/mL was spiked into 3 mL sterile saline, and the MIC (in micrograms per milliliter) was read by Vitek 2 coupled with AST-GN cards. We obtained the breakpoints from CLSI document M100 (15) to determine whether sampled bacterial isolates are susceptible or resistant to the six selected antimicrobials.

### Broth microdilution test.

Since there are no available commercial kits for the newly introduced ceftazidime-avibactam, we used the broth microdilution method in accordance with CLSI standards to determine MICs against ceftazidime-avibactam for bacterial isolates (15). In this test, 10 μL of bacterial suspension at 1.5 × 10^8^ CFU/mL was spiked into 2 mL cation-adjusted Mueller-Hinton (MH) broth. For each isolate, 100 μL bacterium-containing broth was added to microtiter sensitivity test plates and incubated for 18 ± 2 h at 35°C. MICs of ceftazidime-avibactam for both bacterial species were then determined as the lowest concentration that produced complete inhibition of visible growth. The CLSI breakpoints were used to determine the susceptible (≤8 μg/mL) and resistant (≥16 μg/mL) isolates to ceftazidime-avibactam (15). Klebsiella pneumoniae ATCC 700603 and Escherichia coli ATCC 25922 were used as controls.

### EUCAST RAST.

To determine the antimicrobial susceptibility using the EUCAST RAST method, we simulated blood cultures for each bacterial isolate. The concentration of the bacteria used to inoculate blood cultures was standardized to 10 CFU/mL. To do this, we first incubated each bacterial isolate on a Mueller-Hinton agar plate (Oxoid, UK) at 37°C overnight. The bacterial isolates were then diluted to a standard inoculum of 1.5 × 10^8^ CFU/mL and further diluted to 1:10^6^. To make standardized bacterium-containing blood cultures, 2.5 mL of bacterial suspension and 5 mL of sheep blood were spiked into an aerobic bottle (Plus Aerobic/F; Becton Dickinson [BD], USA) containing 30 mL Trypticase soy broth. The bacterium-containing blood cultures were then incubated at 35°C in Bactec (BD, USA).

EUCAST RAST was performed after Bactec flagging of positive bacterium-containing blood cultures. Consistent with the EUCAST guidelines (8), 125 μL of bacterium-containing blood culture was transferred to each 90-mm circular MH agar plate. Discs impregnated with selected antimicrobials were manually placed on plates using a standard distributor. The antimicrobial discs were ordered from Oxoid, including 30 μg amikacin, 5 μg ciprofloxacin, 10 μg imipenem, 5 μg levofloxacin, 10 μg meropenem, and 1.25/23.75 μg trimethoprim-sulfamethoxazole. These disk contents were the same for both EUCAST and CLSI standards, while the disk content of ceftazidime-avibactam (30/20 μg) was consistent with the CLSI standard.

Pictures of the plates were taken after 4, 6, and 8 h of incubation at 35°C, and growth inhibition diameters were manually measured. Since the growth inhibition diameters at 4 h of incubation were not readable for most bacterial isolates, only diameters at 6 and 8 h were recorded for further analyses. Breakpoints for determining susceptible and resistant isolates were obtained from the EUCAST RAST guidelines (8). In addition, bacterial isolates for which the RAST diameter fell in the area of technical uncertainty (ATU; the range between breakpoints of susceptibility and resistance) were identified as isolates that cannot be categorized as either susceptible or resistant (8). Escherichia coli ATCC 25922 was used as the control for the EUCAST RAST method.

### Multiplex PCR for carbapenemase genes.

To characterize the carbapenem-resistant isolates sampled in this study and determine the accuracy of EUCAST RAST, we used multiplex PCR tests to examine the composition of carbapenemase genes in 67 resistant isolates used in this study. In this test, we followed the protocol of Poirel et al. by using multiple pairs of primers to detect the presence of carbapenemase genes in three different families, including class A (*bla*_KPC_ and *bla*_IMI_), class B (*bla*_IMP_, *bla*_VIM_, and *bla*_NDM_), and class D (*bla*_OXA-48_) (16). Carbapenemases in class B can inhibit the activity of ceftazidime-avibactam, while the other two classes cannot (17). Bacterial DNA was obtained from boiling lysis of bacterial suspensions. The PCR amplification was performed as follows: 5 min at 95°C, 38 cycles of 95°C for 30 s, 58°C for 30 s, and 72°C for 45 s, and a final extension at 72°C for 5 min. Amplified PCR products were analyzed by electrophoresis in a 2.5% agarose gel at 110 V for 30 min in a UV transilluminator. The presence of bands at corresponding positions on the gel was used to determine the presence of carbapenemase genes in sampled bacterial isolates.

### Data analyses.

Identification of bacterial susceptibility to the six commonly used antimicrobials by using both traditional and EUCAST RAST methods was compared. We applied the CLSI M52 standard to calculate the rates of categorical agreement (CA), minor errors (mE), major error (ME), and very major error (VME) for each antimicrobial (18). Here, CA refers to percentages of agreement of susceptible and resistant results between commercial and EUCAST RAST methods, mE refers to minor errors where commercial tests show intermediate results while the RAST shows susceptibility or resistance, ME refers to errors where the commercial tests show susceptibility while RAST indicates resistance, and VME refers to errors where the commercial tests show resistance while RAST indicates susceptibility (18). The zone diameters obtained from RAST that fell within the ATU were not counted in categorization. For isolates that were categorized as having ME and VME, a third method, the standard disk diffusion test, was used to test susceptibility (19, 20). Results that were consistent with the standard disk diffusion test were considered more likely to be correct, and these were used to resolve the discrepancy.

To determine operational guidelines for using EUCAST RAST to test the susceptibility of both K. pneumoniae and E. coli to the newly introduced antimicrobial ceftazidime-avibactam (30/20 μg), we used the error rate-bounded method to determine the breakpoints for ceftazidime-avibactam (30/20 μg) (21). The zone diameters and MICs were displayed in scattergrams, and the preliminary RAST breakpoints were drawn as vertical dashed lines.

## RESULTS

### EUCAST RAST efficiently determines the antimicrobial susceptibility in China.

**FIG 1:**
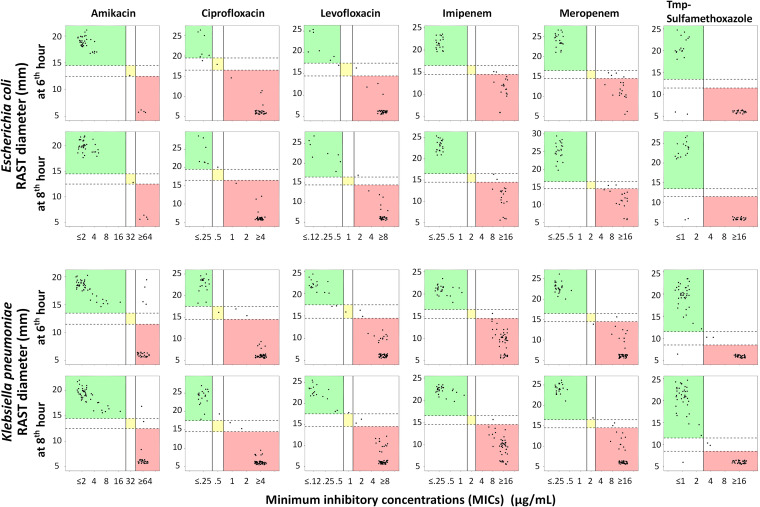
The EUCAST RAST diameters are highly consistent with the MICs from the commercial antimicrobial susceptibility tests for both Escherichia coli (top) and Klebsiella pneumoniae (bottom) at 6 h and 8 h. The vertical solid lines indicate the MIC breakpoints, and the horizontal dashed lines indicate the breakpoints for RAST zone diameters. The green and red areas indicate the categorical agreement of susceptible and resistant, respectively. The isolates in yellow areas had MICs interpreted as intermediate and RAST diameters in the area of technical uncertainty (ATU).

EUCAST RAST was able to determine the susceptibility for most drug-bacterium combinations for K. pneumoniae at 6 h (426/432 [98.6%]) and 8 h (432/432 [100%]) and for E. coli at 6 h (246/246 [100%]) and 8 h (246/246 [100%]), with the overall CA being ≥90% (Fig. 1; Tables 1 and 2). For K. pneumoniae and E. coli, the times for inoculated blood cultures being flagged positive in Bactec were 10.0 ± 0.2 h and 10.8 ± 0.3 h (mean ± standard error), respectively. Low mE rates were observed for K. pneumoniae (0.2% at 6 h; 0.7% at 8 h) and for E. coli (0% at 6 h; 0.4% at 8 h) (Tables 1 and 2). Although low overall ME and VME rates were observed in this study (1.1 and 1.5%, respectively) (Tables 1 and 2), the rates may raise some concerns regarding making clinical decisions for certain antimicrobials. For example, a 11.1% rate of ME was observed for trimethoprim-sulfamethoxazole with E. coli and a 15.4% rate of VME in amikacin tests with K. pneumoniae (Tables 1 and 2). All isolates for which there were ME and VME were tested using the disk diffusion test, and the results were all consistent with those of the commercial tests. The numbers of RAST results that fell within the ATU were 12/426 (2.8%) at 6 h and 10/432 (2.3%) at 8 h for K. pneumoniae and 11/246 (4.5%) at 6 h and 5/246 (2.0%) at 8 h for E. coli. Most of the ATU results were observed in meropenem tests with E. coli (Fig. 1).

**TABLE 1:** Comparison of results between EUCAST RAST and traditional AST methods for Escherichia coli^*a*^

Drug	Time (h)	No. with commercial result			EUCAST RAST rate [no./total (%)] of:			
		S	I	R
					CA	mE	ME	VME
Amikacin	6	36	1	4	40/41 (97.6)	0/41 (0)	0/36 (0)	0/4 (0)
	8				40/41 (97.6)	0/41 (0)	0/36 (0)	0/4 (0)
Ciprofloxacin	6	7	1	33	40/41 (97.6)	0/41 (0)	0/7 (0)	0/33 (0)
	8				39/41 (95.1)	1/41 (2.4)	0/7 (0)	0/33 (0)
Levofloxacin	6	8	0	33	39/41 (95.1)	0/41 (0)	0/8 (0)	0/33 (0)
	8				40/41 (97.6)	0/41 (0)	0/8 (0)	1/33 (3.0)
Imipenem	6	22	0	19	39/41 (95.1)	0/41 (0)	0/22 (0)	0/19 (0)
	8				39/41 (95.1)	0/41 (0)	0/22 (0)	0/19 (0)
Meropenem	6	22	0	19	37/41 (90.2)	0/41 (0)	0/22 (0)	0/19 (0)
	8				39/41 (95.1)	0/41 (0)	0/22 (0)	0/19 (0)
Trimethoprim-sulfamethoxazole	6	18	0	23	39/41 (95.1)	0/41 (0)	2/18 (11.1)	0/23 (0)
	8				39/41 (95.1)	0/41 (0)	2/18 (11.1)	0/23 (0)
Total	6	113	2	131	234/246 (95.1)	0/246 (0)	2/113 (1.8)	0/131 (0)
	8				236/246 (95.9)	1/246 (0.4)	2/113 (1.8)	1/131 (0.8)

a CLSI standard M52 (18) was used to determine the rates of categorical agreement (CA), minor error (mE), major error (ME), and very major error (VME). S, susceptible; I, intermediate; R, resistant.

**TABLE 2:** Comparison of results between EUCAST RAST and traditional AST methods for Klebsiella pneumoniae^*a*^

Drug	Time (h)	No. with commercial result			EUCAST RAST rate [no./total (%)] of:			
		S	I	R
					CA	mE	ME	VME
Amikacin	6	45	0	27	67/71 (94.4)	0/71 (0)	0/45 (0)	4/26 (15.4)
	8				70/72 (97.2)	0/72 (0)	0/45 (0)	1/27 (3.7)
Ciprofloxacin	6	19	1	52	68/71 (95.8)	0/71 (0)	0/19 (0)	0/51 (0)
	8				69/72 (95.8)	1/72 (1.4)	0/19 (0)	0/52 (0)
Levofloxacin	6	19	1	52	66/71 (93.0)	0/71 (0)	0/19 (0)	0/51 (0)
	8				69/72 (95.8)	1/72 (1.4)	0/19 (0)	0/52 (0)
Imipenem	6	25	0	47	70/71 (98.6)	0/71 (0)	0/25 (0)	0/46 (0)
	8				71/72 (98.6)	0/72 (0)	0/25 (0)	0/47 (0)
Meropenem	6	24	1	47	69/71 (97.2)	1/71 (1.4)	0/24 (0)	0/46 (0)
	8				69/72 (95.8)	1/72 (1.4)	0/24 (0)	0/47 (0)
Trimethoprim-sulfamethoxazole	6	38	0	34	68/71 (95.8)	0/71 (0)	1/38 (2.6)	0/33 (0)
	8				69/72 (95.8)	0/72 (0)	1/38 (2.6)	0/34 (0)
Total	6	170	3	259	408/426 (95.8)	1/426 (0.2)	1/170 (0.6)	4/259 (1.5)
	8				417/432 (96.5)	3/432 (0.7)	1/170 (0.6)	1/259 (0.4)

a CLSI standard M52 (18) was used to determine the rates of categorical agreement (CA), minor error (mE), major error (ME), and very major error (VME). S, susceptible; I, intermediate; R, resistant.

### The EUCAST RAST is applicable to the newly introduced antimicrobial ceftazidime-avibactam in China.

**FIG 2:**
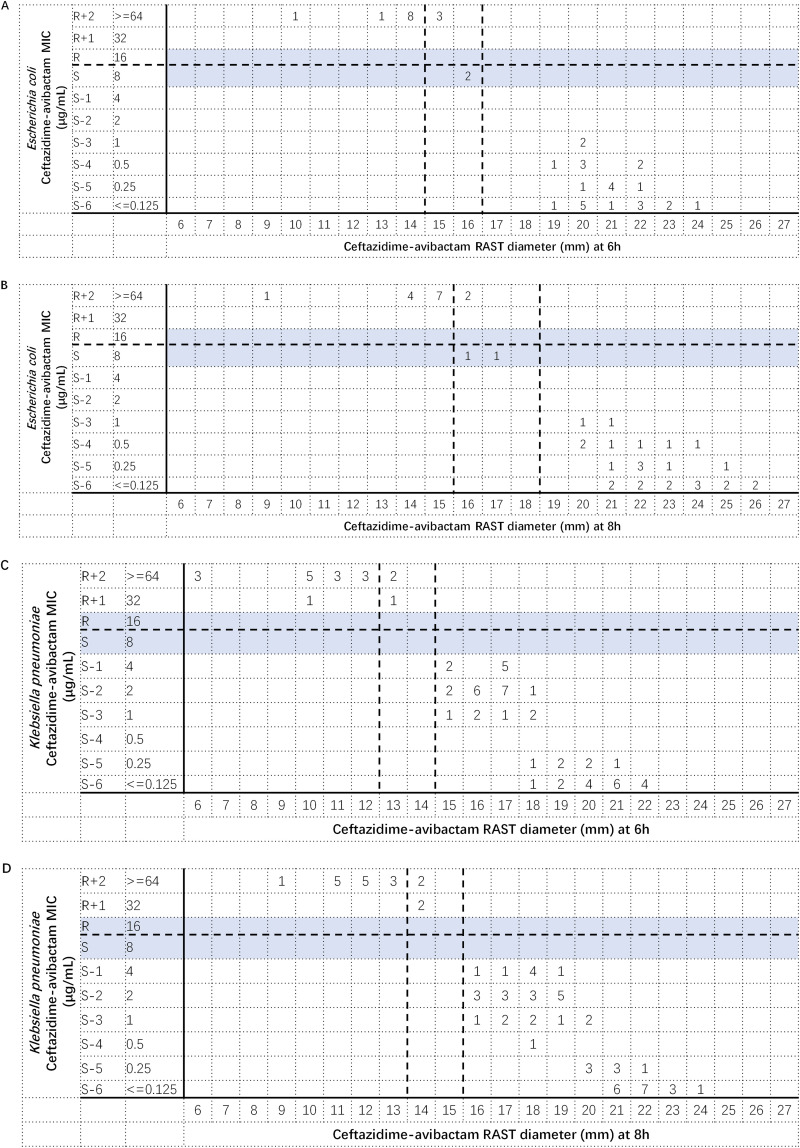
Prediction of EUCAST RAST breakpoints of 30/20 mg ceftazidime-avibactam for Escherichia coli (A and B) and Klebsiella pneumoniae (C and D). Scattergrams comparing the ceftazidime-avibactam MICs and RAST zone diameters. The horizontal and vertical dashed lines indicate the MIC breakpoints and the predicted RAST breakpoints.

In this experiment, we sampled 72 and 43 isolates of K. pneumoniae and E. coli, respectively, to determine the potential breakpoints of resistance and susceptibility for the RAST method. Among all sampled isolates, 18 K. pneumoniae and 14 *E.coli* isolates were ceftazidime-avibactam resistant. We displayed the MICs and RAST diameters in scattergrams for each bacterial at each time and used the MIC breakpoints to determine RAST cutoff values between resistant and susceptible isolates (Fig. 2). The comparisons showed high rates of category agreement with no major or very major discrepancies (CA for E. coli, 88.1% at 6 h and 90.7% at 8 h; CA for K. pneumoniae, 95.8% at 6 h and 94.4% at 8 h). However, the CA rate for E. coli at 6 h did not reach the acceptable threshold criterion (>90%) (18). For both E. coli and K. pneumoniae, 9.3% and 5.6% of isolates fell within the ATU. According to the CLSI M23 guidance (18), the results suggested that the RAST method with an incubation time of 8 h is a reliable method for determining ceftazidime-avibactam susceptibility in E. coli and K. pneumoniae.

### Distribution of carbapenemase genes matches EUCAST RAST results on antimicrobial susceptibility.

In our study, 48 isolates of K. pneumoniae and 19 isolates of E. coli were carbapenem resistant based on the EUCAST RAST. The results from multiplex PCR tests were mostly consistent with the classification by the EUCAST RAST. All isolates of both bacteria that were susceptible to ceftazidime-avibactam have a class A carbapenemase gene, *bla*_KPC_ (Table 3). Isolates that were resistant to ceftazidime-avibactam have two class B carbapenemase genes, *bla*_NDM_ and *bla*_IMP_, and *bla*_NDM_ was present in the majority of resistant isolates (Table 3). Interestingly, among all isolates that fell within the ATU, the majority have class B carbapenemase genes in their genomes (Table 3).

**TABLE 3:** Distribution of antimicrobial resistance genes in carbapenem-resistant *Enterobacterales*^*a*^

Organism	No. of isolates						
	Total	S	ATU			R	
		Class A, KPC	Class A, KPC	Class B, NDM	No carbapenemase	Class B, NDM	Class B, IMP
K. pneumoniae	48	30		2^*b*^	2	12^*c*^	2
E. coli	19	3	1	2	1	12	

a Only *bla*_KPC_, *bla*_IMI_, *bla*_IMP_, *bla*_VIM_, *bla*_NDM_, and *bla*_OXA-48_ were detected in this study. The isolates were arranged based on RAST results at 8 h. S, susceptible; R, resistant.

b One isolate in the ATU coproduced *bla*_KPC_ and *bla*_NDM_.

c One resistant isolate coproduced *bla*_KPC_ and *bla*_NDM_.

## DISCUSSION

In this study, we applied the EUCAST RAST method to determine the antimicrobial susceptibility for two major BSI-related bacterial strains in China. We compared the accuracy of EUCAST RAST with a commonly used commercial method of determining the antimicrobial susceptibility for six commonly used antimicrobials in China. In addition, due to the emerging threat of carbapenem resistance in BSI-causing bacteria and lack of usable commercial kits for rapid evaluation of antimicrobial susceptibility, we developed the breakpoints using the EUCAST RAST method for the recently introduced antimicrobial ceftazidime-avibactam (30/20 μg).

Consistent with results obtained with commercial AST methods, the EUCAST RAST is a reliable method for rapidly determining the antimicrobial susceptibility of BSI-causing bacteria in China. Since RAST was first proposed in 2017, it has been successfully applied to determine the resistance phenotypes of bacterial isolates such as those producing extended-spectrum β-lactamases (ESBL) and carbapenemases and methicillin-resistant Staphylococcus aureus (MRSA) (22). This method is currently used in European countries and has been shown to reduce the diagnostic time to approximately 20 h and thus decrease lengths of stay and mortality in the hospital (9, 23). However, the EUCAST RAST has not been used in China. Current clinical measures in China to determine the antimicrobial susceptibility of BSI-causing bacteria still rely on cultural growth and commercial AST, which usually takes at least 3 days (7). The alternative use of the RAST can significantly reduce this time and have positive clinical impacts on antimicrobial treatments, especially for microbes with potential multidrug-resistant phenotypes (24).

A certain discrepancy has been observed between RAST and commercial methods in determining antimicrobial susceptibility. In our experiments, rates of mE, ME, and VME for E. coli and K. pneumoniae ranged from 0 to 0.7%, 0.8 to 1.8%, and 0.4 to 0.6%, respectively. The error rates are consistent with studies conducted in European countries and some Asian countries, such as Singapore, which had mE rates ranging from 0.5 to 2.2%, ME rates ranging from 0.1 to 5.9%, and VME rates ranging from 0.2 to 1.3% (9, 23, 25, 26). Interestingly, our results showed that most errors in RAST were from tests using amikacin, which has also been observed in other studies using different AST methods. For example, Chandrasekaran et al. used disk diffusion directly from blood culture broth to evaluate the susceptibility to amikacin and found a 23.1% VME (27). In another study using time-kill assays to determine the efficacy of amikacin against another bacterial species in *Enterobacterales*, all four amikacin-susceptible isolates became resistant after 24 h (28). These pieces of evidence suggest the difficulty in determining the susceptibility to amikacin, and amikacin alone may not be an appropriate treatment against *Enterobacterales* infection (29). In addition, our results showed that the uncertainty resulting from the ATU is another reason for discrepancies between RAST and commercial methods, while we observed that only about 2% of isolates fell into the ATU. This percentage is much lower than those in other studies, i.e., 13% to 20% (9, 25, 26). Furthermore, as Jonasson et al. discussed, the presence of bacterial isolates in the ATU has little impact on determining antimicrobial susceptibility and related clinical outcomes when the RAST is used, because the evaluation of antimicrobial susceptibility usually indicates multiple drugs suitable for treatment, and generally, only one or two of them may yield ATU results (25). Taken together, our results show that for the six antimicrobials tested in our study, the RAST is a reliable and practical method to clinically diagnose their antimicrobial susceptibility.

In recent years, the rapid spread of carbapenem-resistant *Enterobacterales* (CRE) has become a serious threat to global health (30). To treat CRE infection, ceftazidime-avibactam was approved in the United States in 2015 and later became the treatment of choice in other countries, including China (17). In our study, we were able to determine preliminary breakpoints for ceftazidime-avibactam (30/20 μg) for the use of RAST with E. coli and K. pneumoniae at both 6 and 8 h. The breakpoints that we predicted can accurately identify susceptible and resistant isolates for the majority of bacteria sampled in China. The multiplex PCR tests showed that the classification of antimicrobial susceptibility in the EUCAST RAST was mostly consistent with the presence of antimicrobial resistance genes in their genomes, which further supported the effectiveness of the preliminary cutoff to determine antimicrobial susceptibility. An interesting observation showed the presence of ceftazidime-avibactam resistance genes in bacterial isolates that fell within the ATU in the EUCAST RAST. Based on MIC measurements, the majority of these isolates in the ATU were resistant to ceftazidime-avibactam, suggesting that EUCAST RAST could leave certain resistant isolates to be identified in diagnostic assays. Therefore, for clinical purposes, it is suggested that for bacterial isolates in the ATU, the decision regarding which antimicrobial agents to use should be carefully considered and may require other data based on molecular methods. A limitation of our study is the lack of further validation for the preliminary breakpoints. As the ceftazidime-avibactam-resistant *Enterobacterales* were still at low prevalence in China and other countries (14, 31), we were not able to collect another batch of ceftazidime-avibactam-resistant bacteria for validation. We recommend that current clinical trials combine the EUCAST RAST and molecular testing to characterize the antimicrobial susceptibility of BSI-causing *Enterobacterales*. Future research with more ceftazidime-avibactam-resistant bacteria can be conducted to determine the effectivity of the breakpoints for ceftazidime-avibactam (30/20 μg).

This is the first study to evaluate the practicality of the EUCAST RAST for BSI-related bacteria in China. Our results showed that RAST is reliable for determining microbial susceptibility to six commonly used antimicrobials in China, including amikacin, ciprofloxacin, levofloxacin, imipenem, meropenem, and trimethoprim-sulfamethoxazole. We also determined potential breakpoints for the recently introduced ceftazidime-avibactam at the concentration used in China and the United States and evaluated the effectiveness by determining the presence of antimicrobial resistance genes. Results of our experiments can be applied to clinically determine the microbial susceptibility of BSI-related bacteria within 8 h and benefit clinical diagnostics for BSI patients.
